# Transcatheter Aortic Valve Implantation in Cancer Patients: A Contemporary Review of the Specific Challenges, the Outcomes, Risk Stratification, and Decision-Making

**DOI:** 10.3390/medicina62061139

**Published:** 2026-06-11

**Authors:** Kalliopi Keramida, Georgios Mavraganis, Constantina Masoura, Konstantinos Aznaouridis, Vasiliki Androutsopoulou, Konstantinos Tsioufis

**Affiliations:** 1 Cardiology Department, General Anticancer Oncological Hospital, Agios Savvas, 11522 Athens, Greece; keramidakalliopi@hotmail.com; 2 Department of Clinical Therapeutics, Alexandra Hospital, School of Medical, National and Kapodistrian University of Athens, 80 Vas. Sofias Str., 11528 Athens, Greece; giomavraganis@gmail.com; 3 Department of Cardiology, General Hospital of Athens “Laiko”, 11527 Athens, Greece; kmasoura@gmail.com; 4 Department of Cardiology, “Hippokration” General Hospital of Athens, 11527 Athens, Greece; conazna@yahoo.com (K.A.); kptsioufis@gmail.com (K.T.); 5 Department of Cardiothoracic Surgery, University Hospital of Larissa, 41110 Larissa, Greece

**Keywords:** transcatheter aortic valve implantation, cancer, frailty, aortic stenosis, cardio-oncology, thoracic radiotherapy, multidisciplinary decision-making

## Abstract

The coexistence of cancer and severe aortic stenosis (AS) is increasing as a result of population aging and substantial improvements in cancer survival. Transcatheter aortic valve implantation (TAVI) has transformed the management of AS; however, patients with active malignancy or a history of cancer remain markedly under-represented in pivotal randomized trials. This under-representation has resulted in persistent uncertainty regarding patient selection, risk stratification, and the expected benefit of TAVI in this growing and clinically heterogeneous population. This review provides a comprehensive and contemporary synthesis of the evidence on TAVI in patients with cancer, integrating cardiovascular (CV), oncologic, and geriatric perspectives. Available data on epidemiological overlap, cancer-specific procedural challenges, and short- and long-term outcomes following TAVI are critically examined, with particular emphasis on distinctions between active cancer and cancer survivorship. Key modifiers of risk and benefit—including prior thoracic radiotherapy, competing thrombotic and bleeding risk, immunosuppression, frailty, sarcopenia, and nutritional status—are discussed in detail. Limitations of conventional surgical risk scores in oncology populations are highlighted, underscoring the need for individualized assessment beyond traditional CV metrics. Across registries and meta-analyses, TAVI is associated with high procedural success and comparable short-term outcomes in patients with and without cancer. Excess mortality observed during mid- and long-term follow-up is driven predominantly by non-CV causes related to malignancy rather than valve-related complications. Importantly, patients with cancer in remission demonstrate outcomes similar to those of non-cancer populations, whereas prognosis in active cancer is strongly influenced by disease stage, biology, and competing risks. Overall, cancer diagnosis alone should not preclude consideration of TAVI. Optimal management requires multidisciplinary, goal-oriented decision-making that integrates oncologic prognosis, functional status, and patients’ priorities. As cancer survivorship continues to expand, prospective studies, integrated risk stratification tools, and closer alignment between cardio-oncology and structural heart programs are essential to guide evidence-based and equitable care.

## 1. Introduction

Transcatheter aortic valve implantation (TAVI) was introduced in 2002 by Alain Cribier in Rouen, France, to offer a minimally invasive treatment option for patients with severe aortic stenosis (AS) deemed unsuitable for surgical valve replacement [1]. Nowadays, TAVI is a well-established therapeutic option for elderly patients with symptomatic severe AS or high surgical risk [2,3]. As survival improves in patients with solid and hematologic malignancies, the number of older cancer survivors is increasing, and since degenerative AS is prevalent in the elderly population, clinicians increasingly encounter patients with cancer (active or prior) and concurrent severe AS [4,5]. These patients may be deemed unsuitable for surgical aortic valve replacement (SAVR) because of prohibitive surgical risk or futility related to frailty, prior thoracic radiotherapy (TRT), severe thrombocytopenia/bleeding risk, or limited life expectancy [3,6]. Historically, patients with active/advanced malignancy were under-represented in pivotal randomized TAVI trials because most trials excluded participants with limited non-cardiac life expectancy (typically <12–24 months) [7,8,9,10,11]. The result is a gap in evidence and an ongoing clinical dilemma: should cancer patients and survivors be offered TAVI, and if so, under what conditions? Important questions remain regarding which cancer patients are most likely to benefit from TAVI and how cardiovascular (CV), oncologic, and geriatric factors should be integrated into clinical decision-making. The aim of this review is to summarize the current evidence regarding TAVI in patients with active cancer and cancer survivorship, highlighting cancer-specific challenges, clinical outcomes, and contemporary approaches to risk stratification. In addition, we discuss existing knowledge gaps and future directions for research in this rapidly evolving field.

## 2. Methodology

This article was conducted as a narrative review aimed at synthesizing the available evidence regarding the challenges, clinical outcomes, and decision-making considerations for TAVI in patients with cancer. Given the heterogeneity of the available evidence and the absence of randomized trials specifically addressing this population, a narrative approach was chosen to integrate findings from different types of studies and provide a clinically oriented overview.

A structured literature search was performed using PubMed/MEDLINE and Scopus to identify relevant publications. The search strategy combined terms related to transcatheter aortic valve intervention with oncology-related terminology, including “TAVI”, “TAVR”, “transcatheter aortic valve implantation”, “aortic stenosis”, “cancer”, “malignancy”, and “cardio-oncology”. Boolean operators were used to combine these terms. The search primarily focused on studies published between 2010 and 2025, reflecting the contemporary era of TAVI practice. Earlier landmark publications were included when necessary to provide historical or conceptual context.

Eligible articles included observational studies, multicenter registries, systematic reviews, meta-analyses, guideline documents, and expert consensus statements addressing TAVI in patients with active cancer or a history of malignancy. Particular attention was given to studies reporting procedural outcomes, long-term survival, cancer-specific procedural challenges, risk stratification, and multidisciplinary decision-making. Articles were screened based on title and abstract for relevance to the topic, followed by full-text evaluation when appropriate. Additional relevant studies were identified through manual review of the reference lists of selected publications. The final selection of studies was based on their methodological quality and relevance to clinical practice.

## 3. Epidemiological Overlap Between Cancer and Aortic Stenosis

An increasing epidemiological overlap exists between cancer and severe AS. Several, mainly retrospective, trials and meta-analyses report that the prevalence of patients with active cancer varies from 3.8% to 8.8% in patients referred for TAVI [12,13,14,15,16,17]. However, patients with a history of cancer and severe AS treated with TAVI have a prevalence of up to 22% in different studies [18], reflecting improved long-term cancer survival. Aging represents the principal driver of both conditions, as both cancer and AS exhibit a marked age-dependent increase. Cancer incidence rises steeply after the sixth decade of life, increasing from approximately 140 per 100,000 in individuals younger than 50 years to >1400 per 100,000 after age 70 and exceeding 2000 per 100,000 in those aged 80 years or older, with nearly 60% of all new cancer diagnoses occurring in adults aged ≥65 years [19]. Degenerative AS is uncommon before midlife, with a prevalence of approximately 0.2% in individuals aged 50–59 years, but increases steeply with advancing age, affecting nearly 10% of octogenarians, among whom approximately 3–5% have severe disease [3,20,21].

Beyond age, cancer and AS share several common risk factors, including chronic inflammation, metabolic disorders such as diabetes and dyslipidemia, smoking, renal dysfunction, and systemic atherosclerotic burden [21,22,23,24,25,26,27,28]. These processes promote both malignant transformation and the initiation and progression of calcific aortic valve disease through overlapping inflammatory, metabolic, and endothelial pathways. In addition, TRT is associated with an increased long-term risk of valvular heart disease, including progressive aortic valve thickening and AS [29,30,31]. These radiation-associated valvular changes typically manifest years to decades after exposure and contribute to the coexistence of cancer and severe AS in contemporary elderly populations [29,32].

## 4. Cancer-Specific Challenges in TAVI

The management of severe AS in patients with cancer presents unique challenges that extend beyond conventional CV risk assessment. Cancer-related factors including oncologic status, treatment history and plan, tumor-related specificities and complications may influence every stage of the TAVI pathway, from patient selection and procedural timing to peri-procedural management and long-term outcomes [33]. In the context of increasing cancer survival and aging populations [34], understanding these challenges is essential to optimize care and avoid both under- and overtreatment.

### 4.1. Heterogeneity of Cancer Types, Stages, and Prognoses

Cancer is not a single entity but a heterogeneous group of diseases with widely variable biological behavior, prognosis, and treatment trajectories [34,35]. Solid tumors and hematologic malignancies differ substantially in their natural history, patterns of progression, and systemic effect [34,35,36]. Even within the same cancer type, prognosis may vary markedly according to stage, molecular profile, and response to therapy [34,35].

For patients with severe AS, this heterogeneity complicates estimation of life expectancy, which remains a key criterion in guideline recommendations for valve intervention [33,37,38]. Historically, a life expectancy of more than one year with acceptable quality of life (QoL) has been required to justify TAVI [37,38]. However, modern oncologic therapies—including targeted agents, immunotherapy, and improved supportive care—have substantially improved survival rates for many malignancies, rendering older prognostic assumptions obsolete [39,40]. Consequently, reliance on cancer diagnosis alone as a marker of futility is increasingly inappropriate.

### 4.2. Hematologic Malignancies

Hematological malignancies, including leukemia, lymphoma, myelodysplastic syndromes, and plasma cell dyscrasias, are increasingly encountered in patients with severe AS evaluated for TAVI [41,42]. Surgical aortic valve replacement (SAVR) is frequently avoided in this population because cardiopulmonary bypass may exacerbate cytopenias, bleeding, infection risk, and immune dysfunction, making TAVI the preferred treatment strategy in selected patients [43,44].

In the largest contemporary nationwide analysis from the United States, hematological malignancy was not associated with increased in-hospital mortality or major peri-procedural complications after TAVI but was linked to higher resource utilization and increased 30- and 90-day non-elective readmissions, driven largely by infectious and cardiovascular causes [41]. Importantly, patients with a history of hematopoietic stem cell transplantation represent a particularly vulnerable subgroup, with a markedly increased risk of in-hospital mortality and respiratory complications after TAVI [41]. Long-term outcome data suggest that, unlike most solid tumors, blood malignancies are associated with reduced mid-term survival after TAVI, even when active cancer does not independently predict early mortality [17,42]. From a procedural standpoint, patients with hematological malignancies frequently meet high-bleeding-risk criteria due to anemia, thrombocytopenia, or platelet dysfunction, necessitating individualized antithrombotic strategies and close collaboration with hematology, as emphasized in the Valve Academic Research Consortium for High Bleeding Risk (VARC-HBR) consensus [45].

In line with contemporary recommendations from the European Society of Cardiology (ESC) valvular heart disease guidelines, the Society for Cardiovascular Angiography and Interventions (SCAI) expert consensus, and recent European national position papers, treatment decisions in this population should be guided by a multidisciplinary heart team, integrating cancer prognosis, bleeding and thrombotic risk, and expected clinical benefit, rather than cancer diagnosis alone [3,46,47,48].

### 4.3. Active Cancer vs. Cancer Survivorship

An important distinction in both clinical practice and research lies between patients with active cancer and those with a history of cancer in remission. Active cancer generally refers to newly diagnosed disease, recurrent or metastatic cancer, or ongoing anticancer therapy, whereas cancer survivorship describes patients with no current evidence of disease following prior treatment. This distinction has important prognostic implications in the context of TAVI.

Patients with a history of cancer in remission generally demonstrate procedural and mid-term outcomes after TAVI comparable to those observed in individuals without cancer, whereas active malignancy is associated with less favorable long-term survival [12]. Importantly, outcomes among patients with active cancer are heterogeneous and strongly influenced by disease stage, biological behavior, and treatment response. However, the absence of standardized definitions for “active cancer” across studies further complicates interpretation of existing data and highlights the need for harmonized classifications in future research.

### 4.4. Thoracic Radiotherapy

TRT is an essential component of treatment for many cancers, including Hodgkin and non-Hodgkin lymphoma, left-sided breast cancer, lung cancer, and esophageal cancer, and is associated with a lifelong increased risk of radiation-induced cardiovascular disease [49].

TRT can result in progressive AV thickening and calcification, leading to radiation-associated AS that typically manifests 10–30 years after exposure, with risk increasing in relation to mean heart dose and radiation dose to cardiac substructures, particularly the valves [30,49]. In Hodgkin lymphoma survivor cohorts, the 30-year cumulative risk of valvular heart disease increased from approximately 3% in patients receiving <30 Gy to 12.4% in those receiving >40 Gy, while relative risk rose stepwise at 31–35 Gy, 36–40 Gy, and >40 Gy [29]. Contemporary cardio-oncology literature increasingly emphasizes cardiac substructure dosimetry, as dose to specific cardiac structures may be more informative than mean heart dose alone. In this context, modeling studies of radiation-induced valvular damage have identified associations with parameters such as left atrial V25 > 63% and left ventricular V30 > 25%, suggesting that chamber- and valve-region exposure may contribute to later valvular dysfunction [50]. Radiation-associated AS often coexists with diffuse coronary artery disease, pericardial fibrosis, myocardial involvement, porcelain aorta, and extensive aorto-mitral curtain calcification, rendering SAVR technically challenging and associated with higher perioperative risk [49].

Observational comparisons therefore support TAVI as the preferred strategy in many patients with radiation-associated AS, with studies demonstrating lower early morbidity and mortality with TAVI compared with SAVR, despite similar long-term survival being limited by non-valvular cardiac disease [51,52]. In a contemporary meta-analysis, patients undergoing TAVI after prior chest radiation had similar short- and mid-term mortality compared with non-irradiated patients, but higher rates of early heart-failure exacerbation and valve reintervention, reflecting the complex radiation-induced cardiac substrate [53].

For the interventional cardiologist, radiation-associated AS presents specific challenges, including severe and asymmetric valvular and left ventricular outflow track calcification, increased conduction disease risk, limited coronary re-access, and higher embolic burden, necessitating meticulous computed tomography-based procedural planning and individualized device selection [49,54].

### 4.5. Competing Thrombotic and Bleeding Risks: Implications for Post-TAVI Antithrombotic Therapy

Cancer is frequently characterized by a paradoxical coexistence of hypercoagulability and increased bleeding risk attributed to tumor biology, systemic inflammation, and anticancer therapies [55,56]. Activation of coagulation pathways increases the risk of venous and arterial thrombosis [57], while bone marrow involvement, chemotherapy, liver dysfunction or renal impairment may predispose to bleeding [58].

Following TAVI, antithrombotic therapy is indicated to reduce the risk of valve thrombosis, cerebrovascular events, and other ischemic complications [38,59,60]. Current post-TAVI antithrombotic therapy favors single antiplatelet therapy (SAPT) as the standard of care to minimize bleeding risk, with dual antiplatelet therapy (DAPT) reserved for selected indications, such as recent coronary stenting or low bleeding risk [3,37,59]. However, in cancer patients, thrombocytopenia, mucosal injury and the need for anticoagulation to treat cancer-associated venous thromboembolism or atrial fibrillation complicates regimen selection [59,61,62]. In patients with cancer and thrombocytopenia, additional pragmatic guidance may be drawn from the 2022 European Hematology Association/European Society of Cardiology recommendations for antithrombotic management in thrombocytopenic cancer patients. These guidelines suggest that low-dose aspirin can generally be maintained in grade 1 thrombocytopenia and used cautiously in grade 2 thrombocytopenia, whereas grade 3 thrombocytopenia usually favors SAPT over DAPT, and grade 4 thrombocytopenia generally warrants withholding antiplatelet therapy until platelet recovery. When DAPT is unavoidable, such as after recent coronary stenting, duration should be minimized as much as clinically feasible and decisions should be individualized in close collaboration with interventional cardiology, oncology/hematology, and, when needed, thrombosis specialists. Importantly, these platelet-based thresholds derive primarily from cancer-thrombocytopenia guidance rather than TAVI-specific studies and should therefore be interpreted as a pragmatic framework rather than definitive evidence for the post-TAVI setting [63]. Nevertheless, cancer-specific data remain limited, particularly regarding optimal duration, drug selection, and management in patients with active malignancy or severe cytopenias, underscoring the need for individualized, multidisciplinary decision-making.

### 4.6. Immunosuppression and Risk of Infection

Immunosuppression related to malignancy or its treatment raises concern for infectious complications following TAVI. Neutropenia, impaired cellular immunity, and frequent healthcare exposure may predispose to bacteremia or endocarditis [64,65], with the latter observed more frequently in older and frailer patients [66]. Although specific data on post-TAVI infection risk in cancer patients are scarce, careful peri-procedural asepsis and close post-procedural monitoring are warranted.

### 4.7. Frailty, Nutritional Status, Cachexia, and Sarcopenia

In patients with severe AS undergoing TAVI, frailty—a state of diminished physiological reserve and increased vulnerability to stressors—represents a major determinant of outcomes, capturing competing risks and reduced physiological reserves that are not adequately reflected by conventional surgical risk scores [67,68]. In non-cancer populations, prospective and trial-based analyses, including the FRAILTY-AVR study and the frailty substudy of the PARTNER trial, have consistently demonstrated that frailty independently predicts mortality, functional decline, and poor QoL outcomes after TAVI, beyond procedural success [67,68].

Beyond frailty assessment alone, comprehensive geriatric assessment (CGA) may provide a more complete evaluation of vulnerability in older patients with cancer. CGA systematically evaluates multiple domains including functional status, comorbidities, cognition, nutritional status, psychological health, and social support, and is widely recommended in geriatric oncology to guide treatment decisions and predict treatment tolerance [69,70]. In patients undergoing AV interventions, multidimensional geriatric assessment has also been shown to improve risk stratification and predict clinical outcomes beyond traditional surgical risk scores [71]. Incorporating CGA into the evaluation of patients with severe AS and cancer may therefore improve individualized decision-making within a multidisciplinary heart team.

Although elderly patients with cancer have a high prevalence of geriatric syndromes and frailty has demonstrated strong prognostic value for QoL, treatment toxicity, and mortality [70,72], evidence supporting its prognostic role specifically in cancer patients undergoing TAVI remains limited. In a retrospective cohort analysis, Kosaraju et al. demonstrated that cancer status—whether active/recent or remote—was not independently associated with 1-year mortality or QoL outcomes following TAVI, whereas frailty was a strong predictor of adverse outcomes in the overall TAVI population [72]. However, when cancer cohorts were analyzed separately, frailty did not retain independent prognostic significance, likely reflecting selection bias and limited sample size, as acknowledged by the authors. Consequently, current guidelines recommend frailty assessment as a key component of heart team decision-making, while recognizing the need for further dedicated research to clarify its prognostic role in cancer patients undergoing TAVI [3].

Cancer-associated cachexia and sarcopenia are prevalent in advanced malignancy and may predict adverse outcomes following CV interventions, including longer intensive care unit stays, higher rates of renal dialysis and care home discharge [73,74,75]. Malnutrition and loss of skeletal muscle mass contribute to frailty, increased susceptibility to infection, and delayed recovery after TAVI [76,77]. Large observational studies and meta-analyses have demonstrated increased mortality and postoperative complications among malnourished or sarcopenic patients undergoing TAVI [78,79]. Assessment of nutritional status and body composition, when feasible, may provide valuable prognostic information beyond traditional risk scores and targeted interventions may improve procedural tolerance in selected patients.

## 5. Clinical Outcomes of TAVI in Cancer Patients

Evidence regarding outcomes of TAVI in patients with cancer is derived primarily from observational studies and multicenter registries, as patients with active malignancy have been traditionally excluded from randomized trials [13]. Despite this limitation, available data provide important insights into procedural safety, short- and long-term outcomes, and the prognostic impact of cancer status. Key studies evaluating TAVI outcomes in patients with cancer are summarized in Table 1.

### 5.1. Procedural Success and Short-Term Outcomes

Across multiple registries, procedural success rates of TAVI in patients with cancer are generally comparable to those observed in non-cancer populations. Early outcomes, including device success, residual aortic regurgitation, and need for conversion to surgery, appear similar between groups [12,15,17,80]. Thirty-day mortality after TAVI is also largely comparable between patients with and without cancer, particularly among those with prior cancer in remission [12,15,17,81]. Early CV mortality attributable to the procedure itself is also not significantly increased in patients with cancer [17].

### 5.2. Procedural Complications

Consistent with the hemostatic abnormalities described before, complications such as active bleeding and transfusion requirements, particularly among patients with active cancer or hematologic malignancies, represent one of the most consistently reported differences between cancer and non-cancer populations undergoing TAVI [17,45,63,82,88]. Rates of other peri-procedural complications such as stroke, acute kidney injury, and CV complications including stroke and myocardial infarction are likewise broadly similar, supporting the technical feasibility and short-term safety of TAVI in carefully selected oncologic patients [17].

Nevertheless, in a large meta-analysis comprising 195,658 patients, although stroke and acute kidney injury rates after TAVI were similar between patients with and without cancer, permanent pacemaker implantation was less frequent in cancer patients [83]. The mechanisms underlying this observation remain unclear. Possible explanations include selection bias, as oncologic patients referred for TAVI may represent a more carefully selected cohort with fewer baseline conduction abnormalities or lower procedural complexity. Survival or attrition bias may also contribute, given the higher competing risk of non-CV mortality in cancer populations, which may reduce the likelihood of detecting later conduction disturbances requiring pacemaker implantation. Differences in procedural characteristics or valve selection may also influence these findings. Further studies are needed to clarify the mechanisms underlying this observation. Infectious complications, including endocarditis, remain relatively uncommon after TAVI [89,90] and registry data have not demonstrated a markedly higher incidence in cancer populations, though selection bias and limited follow-up must be acknowledged [91,92]. Furthermore, overall hospital length of stay after TAVI is similar between cancer and non-cancer patients, with no significant differences reported in discharge timing in most series, although sample sizes and study designs limit definitive conclusions [84,85].

### 5.3. Mid- and Long-Term Survival

Differences in outcomes between cancer and non-cancer patients become more apparent during mid- and long-term follow-up. Multiple observational studies consistently show higher all-cause mortality among cancer patients undergoing TAVI, primarily driven by non-CV deaths related to malignancy progression [12,17,81]. Importantly, CV mortality after TAVI appears broadly similar between cancer and non-cancer patients, suggesting that the valve intervention itself effectively addresses the hemodynamic burden of AS [81,86].

### 5.4. Active vs. History of Cancer: Differential Outcomes

The prognostic impact of cancer status on outcomes after TAVI varies substantially according to disease activity. Patients with a history of cancer in remission consistently demonstrate survival curves comparable to those without cancer, whereas active malignancy—particularly advanced or metastatic disease—is associated with significantly worse long-term survival [12,15,17,33,87]. Certainly, the presence of active cancer is associated with significantly higher 1-year mortality [12], while cancer metastasis is associated with increased mortality after TAVI [80]. Importantly, differences in mortality are primarily driven by non-CV causes related to cancer progression (i.e., advanced stage malignancies) rather than by procedural complications or valve-related failure [13].

However, short-term outcomes following TAVI, including in-hospital and 30-day mortality and key procedural complications such as stroke, acute kidney injury, pacemaker implantation, and major vascular events, appear largely similar irrespective of cancer status, except for a higher rate of major bleeding in patients with active cancer [13,15,83,87,93]. Similarly, cancer survivors with prior TRT have similar 30-day mortality, safety, and efficacy outcomes compared to those without; albeit, they have higher 1-year mortality and congestive heart failure exacerbation [87]. These findings underscore the importance of distinguishing cancer activity when interpreting outcomes and support the use of TAVI in carefully selected patients with active but limited stage or indolent malignancies.

### 5.5. Symptom Relief and Functional Status After TAVI

Data on patient-reported outcomes and functional recovery after TAVI in cancer populations remain limited. Available evidence suggests that many patients experience significant improvement in symptoms and functional status following the procedure albeit less pronounced compared with non-cancer patients [17]. Symptom relief may be particularly meaningful in patients with limited oncologic prognosis, where TAVI may serve a palliative role by reducing heart failure-related burden and improving daily functioning.

### 5.6. Comparison with Surgical Aortic Valve Replacement

Patients with cancer are less frequently referred for SAVR because of perceived operative risk, frailty, and concerns regarding recovery and interaction with cancer therapy [4,84,94,95]. Observational analyses have reported meaningful differences in peri-procedural complications between TAVI and SAVR among patients with cancer. In these studies, TAVI has been associated with lower rates of vascular complications, acute deep venous thrombosis, acute kidney injury, major bleeding and blood transfusion, cardiogenic shock and respiratory complications [95]. Although in-hospital mortality and 30-day readmission rates are similar between TAVI and SAVR [95], TAVI has been associated with higher odds of home discharge compared with SAVR among patients with cancer [84].

In certain oncologic contexts, SAVR may be considered prohibitive, whereas TAVI may still remain a feasible therapeutic option. Examples include patients with prior TRT associated with mediastinal fibrosis or porcelain aorta, those with active malignancy requiring uninterrupted systemic therapy, patients with cancer-related thrombocytopenia or coagulopathy increasing perioperative bleeding risk, and individuals with severe frailty or cancer-associated cachexia in whom prolonged postoperative recovery may be poorly tolerated. Given the absence of randomized data in oncologic populations, treatment decisions should remain individualized and guided by a multidisciplinary heart team, integrating oncologic prognosis, CV risk, procedural feasibility, and patient goals.

## 6. Risk Stratification and Decision-Making in Cancer Patients

Risk stratification and clinical decision-making for TAVI in patients with cancer are inherently complex and require integration of CV, oncologic, functional, and goal-oriented factors, including patient values and treatment priorities. In the context of TAVI and surgical risk assessment, traditional surgical scores [e.g., Society of Thoracic Surgeons (STS) score and EuroSCORE] have limitations because they do not include key cancer-specific factors such as frailty, prior TRT, or other complex comorbidities, and therefore, may be insufficient when used alone [37]. A multidisciplinary, individualized approach is therefore essential.

There is a clear need for integrated risk stratification models combining CV risk, frailty and cancer-specific prognostic factors to improve patient selection and standardize decision-making. Widely used surgical risk scores—i.e., STS score and EuroSCORE II—were not developed or validated in oncology populations and do not incorporate cancer-related variables such as disease stage, activity, or treatment status [37,96]. As a result, these tools may underestimate or misclassify procedural risk in patients with malignancy. Moreover, these scores primarily predict short-term surgical mortality rather than long-term outcomes or QoL, which are often more relevant in patients with cancer [96,97]. They also fail to account for competing risks of non-CV death, a major determinant of prognosis in this population [12,81].

A central challenge in decision-making is determining whether a patient is likely to derive meaningful benefit from TAVI given their overall prognosis. Current guidelines recommend intervention when life expectancy exceeds approximately one year with acceptable QoL; however, this threshold is difficult to apply in oncologic patients [37,38]. Prognostic assessment in cancer is inherently uncertain and depends on tumor biology, stage, molecular characteristics, and treatment responsiveness. Importantly, advances in systemic treatments have significantly improved survival in many malignancies, calling into question the relevance of earlier prognostic assumptions [39,40]. Several observational studies demonstrate that excess mortality after TAVI in cancer patients is predominantly non-CV, underscoring the importance of evaluating competing risks rather than procedural risk alone [12,17]. Tools integrating cancer-specific prognosis with CV risk are currently lacking but represent an important unmet need.

Apart from cancer-specific prognosis, several cancer-specific factors should inform individualized decision-making. These include cancer activity, stage and metastatic burden, expected response to treatment, treatment-related toxicities affecting CV or hematologic systems, and need for uninterrupted cancer therapy.

Current guidelines emphasize that decision-making regarding TAVI should involve a heart team [37]. In patients with cancer, this concept should be expanded to include oncologists, hematologists, geriatricians, and when appropriate, palliative care specialists, constituting a multidisciplinary team (MDT) [98]. Oncologists provide critical insight into disease trajectory, treatment intent, and expected response to therapy, while hematologists contribute to the assessment of cytopenias and thrombotic risk. Geriatricians and palliative care specialists assist in evaluating frailty, functional reserve, and symptom burden. Such collaboration enables more accurate prognostic assessment and coordinated care.

**Figure 1:**
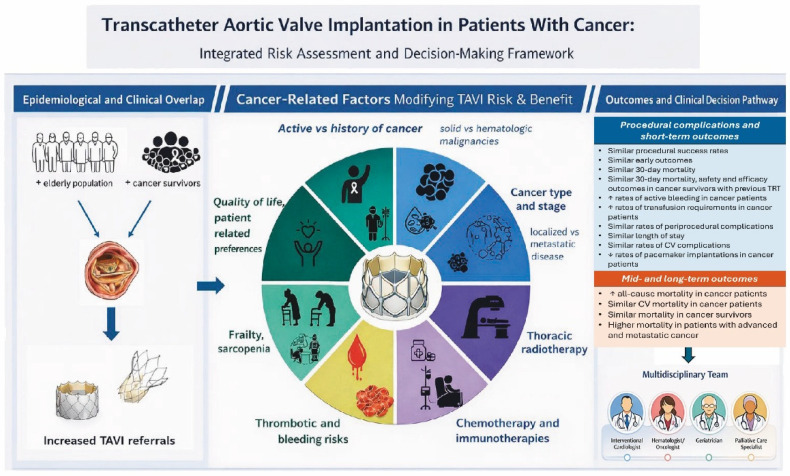
Integrated risk assessment and decision-making framework for transcatheter aortic valve implantation (TAVI) in patients with cancer.

Shared decision-making is central to appropriate care for patients with cancer and severe AS. Discussions should address expected benefits, procedural risks, uncertainty regarding long-term outcomes, and potential interactions with cancer treatment. Patient preferences and priorities—including symptom relief, functional independence, or ability to continue oncologic therapy over longevity—should explicitly guide treatment selection. Within this framework, TAVI may serve both as a disease-modifying therapy and, in selected cases, as a palliative intervention aimed at improving QoL rather than prolonging survival [99,100] (Figure 1).

The above figure illustrates the epidemiological context, cancer-specific modifiers of risk and benefit, and clinical decision pathways relevant to TAVI in patients with active cancer or a history of malignancy. The left panel highlights the increasing epidemiological overlap between cancer survivorship and degenerative aortic stenosis, contributing to rising referrals for TAVI. The central panel depicts key cancer-related factors influencing TAVI outcomes, including cancer activity (active vs. history of cancer), cancer type and stage (solid vs. hematologic malignancies; localized vs. metastatic disease), prior thoracic radiotherapy, exposure to chemotherapy and immunotherapies, thrombotic and bleeding risk, frailty and sarcopenia, and patient-related preferences and quality-of-life considerations. The right panel summarizes procedural complications and short- and mid- to long-term outcomes following TAVI in oncologic populations and emphasizes the role of a multidisciplinary team—including interventional cardiology, oncology/hematology, geriatrics, and palliative care—in individualized, goal-oriented clinical decision-making. Overall, the framework underscores that cancer diagnosis alone should not preclude TAVI and highlights the need for integrated cardiovascular, oncologic, functional, and patient-centered assessment.

## 7. Gaps in Evidence and Future Directions

Despite growing interest in the intersection between structural heart disease and oncology, substantial gaps remain in the evidence guiding TAVI in patients with cancer. Most available data are observational, heterogeneous, and retrospective, limiting causal inference and the development of standardized recommendations. Addressing these limitations is increasingly important as the population of patients living with cancer and CV disease continues to grow (Table 2).

- Under-representation of cancer patients in clinical trials.

Patients with active malignancy have traditionally been excluded from pivotal randomized TAVI trials, resulting in a paucity of high-quality evidence for this population. Consequently, current recommendations rely largely on registry data and retrospective analyses, which are inherently subject to selection bias and residual confounding [13,17]. Future clinical trials should adopt more inclusive eligibility criteria, allowing enrollment of patients with stable or controlled cancer and incorporating predefined subgroup analyses according to cancer type, stage, and activity to improve external validity and relevance to real-world practice.

- Lack of standardized definitions for cancer status and outcomes.

Interpretation of existing studies is further complicated by inconsistent definitions of key oncology-related variables, including “active cancer”, “history of cancer” and “remission” [13,33]. Furthermore, oncologic outcomes are rarely reported in a standardized manner and CV studies often lack detailed information on cancer stage, metastatic burden, or treatment response. Development of consensus definitions and reporting standards would substantially enhance comparability and interpretability across studies.

- Limited integration of oncology-specific prognostic tools.

Current risk stratification for TAVI relies primarily on traditional CV surgical scores such as the STS score and EuroSCORE II, which do not incorporate key cancer-related variables [37,96,97]. Factors such as cancer stage, treatment intent, expected oncologic prognosis, and treatment-related toxicities may significantly influence procedural risk, recovery, and long-term outcomes. Future research should focus on combining oncologic variables with CV risk models and frailty parameters, allowing a more comprehensive assessment of procedural benefit and competing risks in patients with cancer and severe AS.

- Multidimensional geriatric assessment in patient selection.

Older patients with cancer frequently present with complex vulnerability that may not be fully captured by frailty indices alone. Multidimensional geriatric evaluation—including assessment of frailty, nutritional status, sarcopenia, functional capacity, and comorbidities—may improve risk stratification and help identify patients who are most likely to benefit from TAVI. Incorporating geriatric assessment frameworks into the evaluation of oncologic patients with severe AS may therefore support more individualized and clinically meaningful heart team decision-making.

- Insufficient data on optimal antithrombotic strategies.

Antithrombotic management after TAVI in patients with cancer remains particularly challenging due to the coexistence of hypercoagulability and bleeding risk. Although recent trials in the general TAVI population favor simplified antithrombotic regimens [101], cancer patients have been under-represented or excluded from these studies. Dedicated trials are needed to define optimal antithrombotic strategies in this setting, including the role of SAPT, anticoagulation for cancer-associated thrombosis, and management in the presence of thrombocytopenia or active bleeding.

- Long-term outcomes and valve durability.

As cancer survival improves, long-term outcomes after TAVI, including valve durability and late complications, become increasingly relevant. Nevertheless, data on structural valve deterioration, late thrombosis, and reintervention rates in cancer populations are extremely limited, largely due to limited follow-up in existing studies [81]. Prospective longitudinal studies are required, particularly for younger cancer survivors and patients with indolent malignancies.

- Patient-reported outcomes and quality-of-life measures.

Quality of life, symptom burden, and functional independence are often primary goals for patients with cancer and severe AS. However, patient-reported outcomes are inconsistently collected and rarely prioritized as primary endpoints in studies of TAVI involving oncologic patients [17,85], Future investigations should systematically incorporate validated QoL instruments and functional assessments to better capture patient-centered benefits and inform shared decision-making.

- Integration of cardio-oncology and structural heart programs.

The increasing overlap between cancer and structural heart disease highlights the need for integrated cardio-oncology and structural heart programs. Dedicated care pathways may facilitate coordinated referral, standardized assessment of cancer status and frailty, optimized procedural timing relative to oncologic therapy, and harmonized follow-up [102]. Such integrated models also provide a platform for systematic data collection, prospective research, and development of cancer-specific risk stratification tools, ultimately improving evidence generation and care quality in this complex population.

## 8. Limitations

This review has several limitations. First, it was conducted as a narrative review rather than a systematic review, and therefore the literature search and study selection were not performed using formal systematic review methodology. Although a structured search strategy was applied, the possibility of selection bias cannot be completely excluded. Second, the available evidence regarding TAVI in patients with cancer is largely derived from observational studies, retrospective analyses, and registry data. These studies are subject to inherent limitations, including heterogeneity in study design, patient populations, definitions of cancer status, and duration of follow-up. Third, many studies lack detailed oncologic information such as cancer stage, treatment status, and tumor biology, which may significantly influence prognosis and clinical outcomes. Finally, randomized controlled trials specifically addressing patients with active cancer undergoing TAVI remain scarce, limiting the ability to draw definitive conclusions regarding optimal patient selection and management strategies.

## 9. Conclusions

In summary, TAVI may be a feasible and often beneficial treatment option for selected patients with cancer and severe AS. Short-term procedural outcomes are generally comparable to those of non-cancer patients, whereas long-term prognosis is primarily driven by the underlying malignancy rather than CV factors. Cancer history alone should not preclude consideration of TAVI. Optimal management requires individualized, multidisciplinary decision-making that integrates oncologic prognosis, frailty, functional status, and patients’ goals. As cancer survival continues to improve, closer integration between cardio-oncology and structural heart programs, along with prospective research efforts, will be essential to refine risk stratification, guide therapy, and ensure goal-concordant care for this growing patient population.

## Figures and Tables

**Table 1:** Summary of clinical studies evaluating outcomes of transcatheter aortic valve replacement (TAVI) in patients with active cancer or a history of malignancy. Studies are grouped according to study design (observational cohort studies, registries, and systematic reviews or meta-analyses). Arrows indicate the direction of association compared with patients without cancer (↑ higher, ↓ lower, ≈ similar).

First Author	Study Design	N	Study Population	Primary Endpoint	Key Findings	Limitations	Potential Bias
Mangner 2018 [12]	Prospective cohort	1821	Active vs. history vs. no cancer	1-year mortality	↑ 1-year mortality in active cancer	Retrospective single-center design with a relatively small number of patients with active cancer (99 of 1821).	Selection bias due to the retrospective, single-center observational design, in which only patients considered suitable for TAVR were included.
Felix 2024 [13]	Systematic review & meta-analysis	Pooled cohort	Active cancer vs. no cancer	Mortality and VARC outcomes	↑ mortality and bleeding; ≈ procedural complications	Study-level meta-analysis, no subgroup or meta-regression analyses based on cancer type and device used due to incomplete reporting and limited number of included studies.	Detection bias: Patients with cancer are often on anticoagulants or have lower baseline platelets, making bleeding events more likely to be reported.
Bendary 2020 [14]	Systematic review & meta-analysis	5162	Active cancer vs. no cancer	30-day and 1-year mortality	≈ 30-day outcomes; ↑ 1-year mortality	Relies on older studies in which TAVI technology and peri-procedural care were less advanced than current standards.	Publication bias: Small studies showing “no difference” are often not published, which can tilt meta-analysis results toward the “positive” (risk).
Saberian 2025 [15]	Systematic review & meta-analysis	Pooled cohort	Active cancer vs. no cancer	Mortality and complications	≈ short-term outcomes; ↑ 1- and 2-year mortality	Heterogeneity in cancer type, stage, and treatment across the studies included, which may affect the outcomes.	Selection bias due to the inclusion of predominantly observational, non-randomized studies in the meta-analysis, which may influence the pooled outcome estimates.
Landes 2019 [17]	Multinational registry	2744	Active cancer vs. no cancer	Mortality and complications	≈ 30-day mortality; ↑ long-term mortality	As a multi-national registry, there is significant variation in how “active cancer” is defined across different countries and healthcare systems.	Selection bias: Only patients deemed “operable” by high-volume, specialized centers were included, potentially overestimating survival.
Liu 2025 [41]	National cohort study	Large administrative dataset	Hematologic malignancies	Clinical and financial outcomes	↑ complications and resource utilization	Use of an administrative database lacking detailed clinical and oncologic information (e.g., cancer stage, treatment status, and disease activity), which may lead to residual confounding and limit the interpretation of the results.	Selection bias due to the retrospective observational design using an administrative database, in which patient inclusion depends on coding and clinical decision-making rather than randomization.
Biancari 2020 [42]	Retrospective cohort	2130	Active cancer vs. no cancer	Mortality and complications	≈ short-term outcomes; ↑ long-term mortality	High heterogeneity between participating centers regarding patient selection criteria and TAVI techniques.	Reporting/Recall bias: Since this was retrospective, researchers relied on medical records that may have missed minor complications (like small bleeds or delirium), leading to an underestimation of procedural risk.
Kosaraju 2022 [72]	Observational cohort	555	History of cancer	Mortality and quality of life	Frailty predicts mortality and QoL	The subjectivity of frailty assessment tools can vary significantly between different clinicians in a non-standardized setting.	Information/Observer bias: Frailty is often assessed by different clinicians with different “eyes.”
Watanabe 2016 [80]	Multicenter observational cohort	749	Active cancer vs. no cancer	30-day and 1-year mortality	≈ short-term outcomes; ≈ procedural complications	Lacks data on cancer stage, histology, and specific types of active systemic therapy (chemo/radiation).	Selection bias: TAVI may have been preferentially offered to cancer patients perceived as sufficiently fit, potentially biasing outcomes toward more favorable results.
Song 2022 [81]	Systematic review & meta-analysis	pooled cohort	Active cancer vs. no cancer	Mortality and complications	↑ long-term mortality	No published randomized controlled trials were included. Due to short follow-up, the study only analyzed the outcome indexes in the early and medium-term and failed to explore the longer-term prognosis of TAVI.	Heterogeneity bias: High I2 values (statistical inconsistency) often occur because of different follow-up durations across pooled studies.
Aikawa 2023 [82]	Nationwide registry	122,573	Active cancer vs. no cancer	Mortality and readmissions	≈ in-hospital mortality; ↑ readmissions	Nationwide registries often lack “granular” data, such as left ventricular ejection fraction (LVEF), surgical risk scores or specific anatomical challenges (e.g., porcelain aorta).	Confounding by indication: Patients with cancer might be readmitted more often not due to TAVI failure, but due to cancer-related complications (infection, anemia).
Osawa 2024 [83]	Systematic review & meta-analysis	Pooled cohort	Active or previous cancer	Mortality and complications	↑ long-term mortality	Pools “active” and “previous” cancer together; this significantly obscures the actual risk of patients currently undergoing therapy.	Dilution bias: Including patients with a history of cancer (cured) “dilutes” the mortality signal compared to those with truly active, advanced disease.
Guha 2020 [84]	National database study	63,352	Active cancer vs. no cancer	Procedural outcomes	≈ short-term mortality; ↑ readmissions	Relies on ICD-10 codes, which often lack details on cancer severity, current chemotherapy status, or biological frailty markers.	Misclassification bias: Coding errors in large databases can lead to patients with a “history of cancer” being labeled as “active cancer,” or vice versa.
Kojima 2022 [85]	Multicenter cohort	1114	Active cancer vs. no cancer	Survival outcomes	↑ mortality in metastatic cancer	The number of patients with active malignancy and the follow-up period were limited.	Survival bias: Patients with metastatic cancer who live long enough to be referred for, worked up for, and receive a TAVI are already “biological survivors.”
Diaz-Arocutipa 2021 [86]	Systematic review & meta-analysis	255,840	Active or previous cancer	Mortality and complications	↑ long-term mortality; ≈ procedural complications	The massive N (255,840) is driven by administrative databases, which dilutes the precision of “active” vs. “history of” cancer definitions.	Aggregation bias: By pooling very large registry data with small high-quality cohorts, the registry “noise” may drown out specific clinical insights.
Lind 2020 [87]	Single-center cohort	892	Active cancer vs. no cancer	Mortality and complications	↑ mortality in cancer patients	Single-center study with a relatively small sample size, limiting the generalizability of the findings to broader populations. Long period of recruitment, during which guidelines for TAVI have changed.	Detection bias: Because cancer patients are monitored more closely by oncologists (scans, blood tests), complications are more likely to be “detected” in this group compared to the general population.

Abbreviations: TAVI = transcatheter aortic valve implantation; TAVR = transcatheter aortic valve replacement.

**Table 2:** Key priorities for future research in transcatheter aortic valve implantation (TAVI) among patients with active cancer and cancer survivors.

Key Priorities for Future Research Regarding TAVI in Cancer Patients and Survivors:
Prospective dedicated studies and pragmatic trials including patients with active cancer and cancer survivors undergoing TAVI. Standardized definitions of cancer status, including active cancer, recent cancer, and cancer survivorship, together with consistent reporting of oncologic outcomes. Development of integrated cardiovascular–oncologic risk stratification models incorporating cancer stage, treatment status, and competing mortality risk. Incorporation of multidimensional geriatric assessment (including frailty, nutritional status, and sarcopenia) into patient-selection frameworks. Optimization of antithrombotic strategies after TAVI in patients with cancer, particularly in the presence of thrombocytopenia or competing thrombotic risk. Long-term assessment of valve durability and late cardiovascular complications in cancer survivors. Systematic incorporation of patient-reported outcomes and quality-of-life measures. Development of dedicated cardio-oncology–structural heart collaboration frameworks. Evaluation of multidisciplinary care models integrating cardiology, oncology, geriatrics, and palliative care.

## Data Availability

No new data were created or analyzed in this study. Data sharing is not applicable to this article.

## References

[1] Cribier A., Eltchaninoff H., Bash A., Borenstein N., Tron C., Bauer F., Derumeaux G., Anselme F., Laborde F., Leon M.B. (2002). Percutaneous transcatheter implantation of an aortic valve prosthesis for calcific aortic stenosis: First human case description. Circulation.

[2] Sundt T.M., Jneid H. (2021). Guideline Update on Indications for Transcatheter Aortic Valve Implantation Based on the 2020 American College of Cardiology/American Heart Association Guidelines for Management of Valvular Heart Disease. JAMA Cardiol..

[3] Praz F., Borger M.A., Lanz J., Marin-Cuartas M., Abreu A., Adamo M., Marsan N.A., Barili F., Bonaros N., Cosyns B. (2025). 2025 ESC/EACTS Guidelines for the management of valvular heart disease: Developed by the task force for the management of valvular heart disease of the European Society of Cardiology (ESC) and the European Association for Cardio-Thoracic Surgery (EACTS). Eur. Heart J..

[4] Santangelo G., Moscardelli S., Barbieri L., Faggiano A., Carugo S., Faggiano P. (2023). Aortic Valve Stenosis and Cancer: Problems of Management. J. Clin. Med..

[5] Giza D.E., Iliescu G., Hassan S., Marmagkiolis K., Iliescu C. (2017). Cancer as a Risk Factor for Cardiovascular Disease. Curr. Oncol. Rep..

[6] Abruzzo A.R., McGurk S., Tolis G., Aranki S., Sabe A., Cunningham M.J., Nohria A., Itoh A. (2025). Outcomes of surgical valve replacements for radiation-induced valvulopathy. JTCVS Open.

[7] Smith C.R., Leon M.B., Mack M.J., Miller D.C., Moses J.W., Svensson L.G., Tuzcu E.M., Webb J.G., Fontana G.P., Makkar R.R. (2011). Transcatheter versus surgical aortic-valve replacement in high-risk patients. N. Engl. J. Med..

[8] Leon M.B., Smith C.R., Mack M.J., Makkar R.R., Svensson L.G., Kodali S.K., Thourani V.H., Tuzcu E.M., Miller D.C., Herrmann H.C. (2016). Transcatheter or Surgical Aortic-Valve Replacement in Intermediate-Risk Patients. N. Engl. J. Med..

[9] Mack M.J., Leon M.B., Thourani V.H., Makkar R., Kodali S.K., Russo M., Kapadia S.R., Malaisrie S.C., Cohen D.J., Pibarot P. (2019). Transcatheter Aortic-Valve Replacement with a Balloon-Expandable Valve in Low-Risk Patients. N. Engl. J. Med..

[10] Kapadia S.R., Leon M.B., Makkar R.R., Tuzcu E.M., Svensson L.G., Kodali S., Webb J.G., Mack M.J., Douglas P.S., Thourani V.H. (2015). 5-year outcomes of transcatheter aortic valve replacement compared with standard treatment for patients with inoperable aortic stenosis (PARTNER 1): A randomised controlled trial. Lancet.

[11] Reardon M.J., Adams D.H., Kleiman N.S., Yakubov S.J., Coselli J.S., Deeb G.M., Gleason T.G., Lee J.S., Hermiller J.B., Chetcuti S. (2015). 2-Year Outcomes in Patients Undergoing Surgical or Self-Expanding Transcatheter Aortic Valve Replacement. J. Am. Coll. Cardiol..

[12] Mangner N., Woitek F.J., Haussig S., Holzhey D., Stachel G., Schlotter F., Höllriegel R., Mohr F.W., Schuler G., Linke A. (2018). Impact of active cancer disease on the outcome of patients undergoing transcatheter aortic valve replacement. J. Interv. Cardiol..

[13] Felix N., Nogueira A., Carvalho P.E.P., Costa T.A., Tramujas L., Generoso G., Feldman S., Garot P., de Farias M.D.C.A.D. (2024). Outcomes of patients with active cancer after transcatheter aortic valve replacement: An updated meta-analysis. Cardio-Oncology.

[14] Bendary A., Ramzy A., Bendary M., Salem M. (2020). Transcatheter aortic valve replacement in patients with severe aortic stenosis and active cancer: A systematic review and meta-analysis. Open Heart.

[15] Saberian P., Contreras R., Gurram A., Nasrollahizadeh A., Keetha N.R., Nguyen A.L., Nayak S.S., Keivanlou M., Hashemi M., Amini-Salehi E. (2025). Clinical Outcomes and Prognostic Implications of TAVR in Patients With Active Cancer: A Meta-Analysis. Clin. Cardiol..

[16] Noguchi M., Tabata M., Ito J., Kato N., Obunai K., Watanabe H., Yashima F., Watanabe Y., Naganuma T., Yamawaki M. (2024). Midterm outcomes of transcatheter aortic valve replacement in patients with active cancer. Open Heart.

[17] Landes U., Iakobishvili Z., Vronsky D., Zusman O., Barsheshet A., Jaffe R., Jubran A., Yoon S.-H., Makkar R.R., Taramasso M. (2019). Transcatheter Aortic Valve Replacement in Oncology Patients With Severe Aortic Stenosis. JACC Cardiovasc. Interv..

[18] Ahsan U., Naz S., Anum A., Unum A., Hamza R.M., Qasim R.M., Taaruf A., Khan N. (2024). Outcomes and Adverse Effects of Transcatheter Aortic Valve Replacement (TAVR) in Cancer Patients: A Meta-Analysis. Cureus.

[19] Siegel R.L., Kratzer T.B., Giaquinto A.N., Sung H., Jemal A. (2025). Cancer statistics, 2025. CA Cancer J. Clin..

[20] Osnabrugge R.L., Mylotte D., Head S.J., Van Mieghem N.M., Nkomo V.T., LeReun C.M., Bogers A.J.J.C., Piazza N., Kappetein A.P. (2013). Aortic stenosis in the elderly: Disease prevalence and number of candidates for transcatheter aortic valve replacement: A meta-analysis and modeling study. J. Am. Coll. Cardiol..

[21] Otto C.M., Prendergast B. (2014). Aortic-valve stenosis--from patients at risk to severe valve obstruction. N. Engl. J. Med..

[22] Giovannucci E., Harlan D.M., Archer M.C., Bergenstal R.M., Gapstur S.M., Habel L.A., Pollak M., Regensteiner J.G., Yee D. (2010). Diabetes and cancer: A consensus report. Diabetes Care.

[23] Hanahan D., Weinberg R.A. (2011). Hallmarks of cancer: The next generation. Cell.

[24] Yuan F., Wen W., Jia G., Long J., Shu X.O., Zheng W. (2023). Serum Lipid Profiles and Cholesterol-Lowering Medication Use in Relation to Subsequent Risk of Colorectal Cancer in the UK Biobank Cohort. Cancer Epidemiol. Biomark. Prev..

[25] Wong G., Hayen A., Chapman J.R., Webster A.C., Wang J.J., Mitchell P., Craig J.C. (2009). Association of CKD and cancer risk in older people. J. Am. Soc. Nephrol..

[26] Thanassoulis G., Campbell C.Y., Owens D.S., Smith J.G., Smith A.V., Peloso G.M., Kerr K.F., Pechlivanis S., Budoff M.J., Harris T.B. (2013). Genetic associations with valvular calcification and aortic stenosis. N. Engl. J. Med..

[27] Rattazzi M., Bertacco E., Del Vecchio A., Puato M., Faggin E., Pauletto P. (2013). Aortic valve calcification in chronic kidney disease. Nephrol. Dial. Transplant..

[28] Stewart B.F., Siscovick D., Lind B.K., Gardin J.M., Gottdiener J.S., Smith V.E., Kitzman D.W., Otto C.M. (1997). Clinical factors associated with calcific aortic valve disease. Cardiovascular Health Study. J. Am. Coll. Cardiol..

[29] Cutter D.J., Schaapveld M., Darby S.C., Hauptmann M., van Nimwegen F.A., Krol A.D., Janus C.P., van Leeuwen F.E., Aleman B.M. (2015). Risk of valvular heart disease after treatment for Hodgkin lymphoma. J. Natl. Cancer Inst..

[30] Lee C., Hahn R.T. (2023). Valvular Heart Disease Associated With Radiation Therapy: A Contemporary Review. Struct. Heart J. Heart Team.

[31] Belzile-Dugas E., Eisenberg M.J. (2021). Radiation-Induced Cardiovascular Disease: Review of an Underrecognized Pathology. J. Am. Heart Assoc..

[32] Hull M.C., Morris C.G., Pepine C.J., Mendenhall N.P. (2003). Valvular dysfunction and carotid, subclavian, and coronary artery disease in survivors of hodgkin lymphoma treated with radiation therapy. JAMA.

[33] Leedy D., Elison D.M., Farias F., Cheng R., McCabe J.M. (2023). Transcatheter aortic valve intervention in patients with cancer. Heart (Br. Card. Soc.).

[34] Sung H., Ferlay J., Siegel R.L., Laversanne M., Soerjomataram I., Jemal A., Bray F. (2021). Global Cancer Statistics 2020: GLOBOCAN Estimates of Incidence and Mortality Worldwide for 36 Cancers in 185 Countries. CA Cancer J. Clin..

[35] Siegel R.L., Miller K.D., Wagle N.S., Jemal A. (2023). Cancer statistics, 2023. CA Cancer J. Clin..

[36] Khorana A.A., Francis C.W., Culakova E., Fisher R.I., Kuderer N.M., Lyman G.H. (2006). Thromboembolism in hospitalized neutropenic cancer patients. J. Clin. Oncol..

[37] Vahanian A., Beyersdorf F., Praz F., Milojevic M., Baldus S., Bauersachs J., Capodanno D., Conradi L., De Bonis M., De Paulis R. (2022). 2021 ESC/EACTS Guidelines for the management of valvular heart disease. Eur. Heart J..

[38] Otto C.M., Nishimura R.A., Bonow R.O., Carabello B.A., Erwin J.P., Gentile F., Jneid H., Krieger E.V., Mack M., McLeod C. (2021). 2020 ACC/AHA Guideline for the Management of Patients With Valvular Heart Disease: Executive Summary: A Report of the American College of Cardiology/American Heart Association Joint Committee on Clinical Practice Guidelines. Circulation.

[39] Shahid K., Khalife M., Dabney R., Phan A.T. (2019). Immunotherapy and targeted therapy-the new roadmap in cancer treatment. Ann. Transl. Med..

[40] Vanneman M., Dranoff G. (2012). Combining immunotherapy and targeted therapies in cancer treatment. Nat. Rev. Cancer.

[41] Liu Z., Aguayo E., Porter G., Ali K., Zinoviev R., Benharash P. (2025). Association of hematological malignancies with clinical and financial outcomes following transcatheter aortic valve replacement. Clinics.

[42] Biancari F., Dahlbacka S., Juvonen T., Virtanen M.P.O., Maaranen P., Jaakkola J., Laakso T., Niemelä M., Tauriainen T., Vento A. (2020). Favorable outcome of cancer patients undergoing transcatheter aortic valve replacement. Int. J. Cardiol..

[43] Balanescu S.M., Balanescu D.V., Donisan T., Yang E.H., Palaskas N., Lopez-Mattei J., Hassan S., Kim P., Cilingiroglu M., Marmagkiolis K. (2019). The Onco-cardiologist Dilemma: To Implant, to Defer, or to Avoid Transcatheter Aortic Valve Replacement in Cancer Patients with Aortic Stenosis?. Curr. Cardiol. Rep..

[44] Sarı C., Ayhan H., Baştuğ S., Kasapkara H.A., Karaduman B.D., Aslan A.N., Özen M.B., Bilen E., Bayram N.A., Keleş T. (2015). Transcatheter aortic valve implantation in the presence of hematologic malignancies. Turk. Kardiyol. Dern. Ars..

[45] Garot P., Morice M.C., Angiolillo D.J., Cabau J.R., Park D.W., Van Mieghem N.M., Collet J.-P., Leon M.B., Sengottuvelu G., Neylon A. (2024). Defining high bleeding risk in patients undergoing transcatheter aortic valve implantation: A VARC-HBR consensus document. EuroIntervention.

[46] Iliescu C.A., Grines C.L., Herrmann J., Yang E.H., Cilingiroglu M., Charitakis K., Hakeem A., Toutouzas K.P., Leesar M.A., Marmagkiolis K. (2016). SCAI Expert consensus statement: Evaluation, management, and special considerations of cardio-oncology patients in the cardiac catheterization laboratory (endorsed by the cardiological society of india, and sociedad Latino Americana de Cardiologıa intervencionista). Catheter. Cardiovasc. Interv..

[47] Nobre Menezes M., Tavares da Silva M., Magalhães A., Melica B., Toste J.C., Calé R., Almeida M., Fiuza M., de Oliveira E.I. (2024). Interventional cardiology in cancer patients: A position paper from the Portuguese Cardiovascular Intervention Association and the Portuguese Cardio-Oncology Study Group of the Portuguese Society of Cardiology. Rev. Port. Cardiol..

[48] Cequier Á., Pérez de Prado A., Moreno R., Cosín-Sales J., López de Sá E., Evangelista A., Bueno H., Anguita M. (2019). Percutaneous cardiological intervention and cardiac surgery: Patient-centered care. Position statement of the Spanish Society of Cardiology. Rev. Esp. Cardiol..

[49] Bergom C., Bradley J.A., Ng A.K., Samson P., Robinson C., Lopez-Mattei J., Mitchell J.D. (2021). Past, Present, and Future of Radiation-Induced Cardiotoxicity: Refinements in Targeting, Surveillance, and Risk Stratification. JACC Cardio Oncol..

[50] Cella L., Oh J.H., Deasy J.O., Palma G., Liuzzi R., D’avino V., Conson M., Picardi M., Salvatore M., Pacelli R. (2015). Predicting radiation-induced valvular heart damage. Acta Oncol..

[51] Agrawal N., Kattel S., Waheed S., Kapoor A., Singh V., Sharma A., Page B.J., Attwood K.M., Iyer V., Pokharel S. (2019). Clinical Outcomes after Transcatheter Aortic Valve Replacement in Cancer Survivors Treated with Ionizing Radiation. Cardio-Oncology.

[52] Patil S., Pingle S.R., Shalaby K., Kim A.S. (2022). Mediastinal irradiation and valvular heart disease. Cardio-Oncology.

[53] Mitchell J.D., Cehic D.A., Morgia M., Bergom C., Toohey J., Guerrero P.A., Ferencik M., Kikuchi R., Carver J.R., Zaha V.G. (2021). Cardiovascular Manifestations from Therapeutic Radiation: A Multidisciplinary Expert Consensus Statement From the International Cardio-Oncology Society. JACC Cardio Oncol..

[54] Allen C.J., Patterson T., Prendergast B., Roberts-Thompson R.L., Rajani R., Redwood S.R. (2020). Simultaneous Transcatheter Double Valve Treatment of Mediastinal Radiation-Induced Severe Calcific Aortic and Mitral Stenosis. JACC Case Rep..

[55] Buller H.R., van Doormaal F.F., van Sluis G.L., Kamphuisen P.W. (2007). Cancer and thrombosis: From molecular mechanisms to clinical presentations. J. Thromb. Haemost..

[56] Sud R., Khorana A.A. (2009). Cancer-associated thrombosis: Risk factors, candidate biomarkers and a risk model. Thromb. Res..

[57] Franchini M., Tufano A., Casoria A., Coppola A. (2021). Arterial Thrombosis in Cancer Patients: An Update. Semin. Thromb. Hemost..

[58] Frere C., Font C., Esposito F., Crichi B., Girard P., Janus N. (2022). Incidence, risk factors, and management of bleeding in patients receiving anticoagulants for the treatment of cancer-associated thrombosis. Support. Care Cancer.

[59] Ten Berg J., Sibbing D., Rocca B., Van Belle E., Chevalier B., Collet J.P., Dudek D., Gilard M., Gorog D.A., Grapsa J. (2021). Management of antithrombotic therapy in patients undergoing transcatheter aortic valve implantation: A consensus document of the ESC Working Group on Thrombosis and the European Association of Percutaneous Cardiovascular Interventions (EAPCI), in collaboration with the ESC Council on Valvular Heart Disease. Eur. Heart J..

[60] Nijenhuis V.J., Brouwer J., Søndergaard L., Collet J.P., Grove E.L., Ten Berg J.M. (2019). Antithrombotic therapy in patients undergoing transcatheter aortic valve implantation. Heart.

[61] Cereda A., Lucreziotti S., Franchina A.G., Laricchia A., De Regibus V., Conconi B., Carlà M., Spangaro A., Rocchetti M., Ponti L. (2023). Systematic Review and Meta-Analysis of Oral Anticoagulant Therapy in Atrial Fibrillation Cancer Patients. Cancers.

[62] Naidu M.U., Ramana G.V., Rani P.U., Mohan I.K., Suman A., Roy P. (2004). Chemotherapy-induced and/or radiation therapy-induced oral mucositis--complicating the treatment of cancer. Neoplasia.

[63] Falanga A., Leader A., Ambaglio C., Bagoly Z., Castaman G., Elalamy I., Lecumberri R., Niessner A., Pabinger I., Szmit S. (2022). EHA Guidelines on Management of Antithrombotic Treatments in Thrombocytopenic Patients With Cancer. Hemasphere.

[64] Rapoport B.L. (2011). Management of the cancer patient with infection and neutropenia. Semin. Oncol..

[65] Seo S.K., Liu C., Dadwal S.S. (2021). Infectious Disease Complications in Patients with Cancer. Crit. Care Clin..

[66] Strange J.E., Østergaard L., Køber L., Bundgaard H., Iversen K., Voldstedlund M., Gislason G.H., Olesen J.B., Fosbøl E.L. (2023). Patient Characteristics, Microbiology, and Mortality of Infective Endocarditis After Transcatheter Aortic Valve Implantation. Clin. Infect. Dis..

[67] Afilalo J., Lauck S., Kim D.H., Lefèvre T., Piazza N., Lachapelle K., Martucci G., Lamy A., Labinaz M., Peterson M.D. (2017). Frailty in Older Adults Undergoing Aortic Valve Replacement: The FRAILTY-AVR Study. J. Am. Coll. Cardiol..

[68] Green P., Arnold S.V., Cohen D.J., Kirtane A.J., Kodali S.K., Brown D.L., Rihal C.S., Xu K., Lei Y., Hawkey M.C. (2015). Relation of frailty to outcomes after transcatheter aortic valve replacement (from the PARTNER trial). Am. J. Cardiol..

[69] Mohile S.G., Velarde C., Hurria A., Magnuson A., Lowenstein L., Pandya C., O’Donovan A., Gorawara-Bhat R., Dale W. (2015). Geriatric Assessment-Guided Care Processes for Older Adults: A Delphi Consensus of Geriatric Oncology Experts. J. Natl. Compr. Cancer Netw..

[70] Wildiers R.A., Mohile S., Repetto L., Van Leeuwen B., Milisen K., Hurria A. (2014). International Society of Geriatric Oncology consensus on geriatric assessment in older patients with cancer. J. Clin. Oncol..

[71] Stortecky S., Schoenenberger A.W., Moser A., Kalesan B., Jüni P., Carrel T., Bischoff S., Schoenenberger C.M., Stuck A.E., Windecker S. (2012). Evaluation of multidimensional geriatric assessment as a predictor of mortality and cardiovascular events after transcatheter aortic valve implantation. JACC Cardiovasc. Interv..

[72] Kosaraju N., Wu P., Leng M., Bolano M., Rafique A.M., Shen J., Satou N., Huchting J., Goldwater D., Aksoy O. (2022). Impact of frailty on mortality and quality of life in patients with a history of cancer undergoing transcatheter aortic valve replacement. Clin. Cardiol..

[73] Damluji A.A., Alfaraidhy M., AlHajri N., Rohant N.N., Kumar M., Al Malouf C., Bahrainy S., Kwak M.J., Batchelor W.B., Forman D.E. (2023). Sarcopenia and Cardiovascular Diseases. Circulation.

[74] Zhang Y., Zhang J., Zhan Y., Pan Z., Liu Q., Yuan W. (2024). Sarcopenia Is a Prognostic Factor of Adverse Effects and Mortality in Patients With Tumour: A Systematic Review and Meta-Analysis. J. Cachexia Sarcopenia Muscle.

[75] Ansaripour A., Arjomandi Rad A., Koulouroudias M., Angouras D., Athanasiou T., Kourliouros A. (2023). Sarcopenia Adversely Affects Outcomes following Cardiac Surgery: A Systematic Review and Meta-Analysis. J. Clin. Med..

[76] Muresan B.T., Núñez-Abad M., Artero A., Rios J., Cunquero-Tomás A., Iranzo V., Garrido J., Jiménez-Portilla A., Herrero C.C., Juan C.J.S. (2022). Relation of Malnutrition and Nosocomical Infections in Cancer Patients in Hospital: An Observational Study. J. Nutr. Metab..

[77] Bertschi D., Kiss C.M., Schoenenberger A.W., Stuck A.E., Kressig R.W. (2021). Sarcopenia in Patients Undergoing Transcatheter Aortic Valve Implantation (TAVI): A Systematic Review of the Literature. J. Nutr. Health Aging.

[78] Yang Y.W., Pan P., Xia X., Zhou Y.W., Ge M.L. (2023). Prognostic value of sarcopenia in older adults with transcatheter aortic valve implantation: A systematic review and meta-analysis. Arch. Gerontol. Geriatr..

[79] He J. (2025). Prognostic value of sarcopenia in aortic valve replacement: A systematic review and meta-analysis. Front. Nutr..

[80] Watanabe Y., Kozuma K., Hioki H., Kawashima H., Nara Y., Kataoka A., Shirai S., Tada N., Araki M., Takagi K. (2016). Comparison of Results of Transcatheter Aortic Valve Implantation in Patients With Versus Without Active Cancer. Am. J. Cardiol..

[81] Song Y., Wang Y., Wang Z., Xu C., Dou J., Jiang T. (2022). Comparing Clinical Outcomes on Oncology Patients With Severe Aortic Stenosis Undergoing Transcatheter Aortic Valve Implantation: A Systematic Review and Meta-Analysis. Front. Cardiovasc. Med..

[82] Aikawa T., Kuno T., Malik A.H., Briasoulis A., Kolte D., Kampaktsis P.N., Latib A. (2023). Transcatheter Aortic Valve Replacement in Patients With or Without Active Cancer. J. Am. Heart Assoc..

[83] Osawa T., Tajiri K., Hoshi T., Ieda M., Ishizu T. (2024). Impact of cancer in patients with aortic stenosis undergoing transcatheter aortic valve replacement: A systematic review and meta-analysis. Int. J. Cardiol. Heart Vasc..

[84] Guha A., Dey A.K., Arora S., Cavender M.A., Vavalle J.P., Sabik J.F., Jimenez E., Jneid H., Addison D. (2020). Contemporary Trends and Outcomes of Percutaneous and Surgical Aortic Valve Replacement in Patients With Cancer. J. Am. Heart Assoc..

[85] Kojima Y., Higuchi R., Hagiya K., Saji M., Takamisawa I., Iguchi N., Takanashi S., Doi S., Okazaki S., Sato K. (2022). Prognosis of patients with active cancer undergoing transcatheter aortic valve implantation: An insight from Japanese multicenter registry. Int. J. Cardiol. Heart Vasc..

[86] Diaz-Arocutipa C., Torres-Valencia J., Zavaleta-Camacho G., Vicent L. (2021). Association Between Previous or Active Cancer and Clinical Outcomes in TAVR Patients: A Systematic Review and Meta-Analysis of 255,840 Patients. Front. Cardiovasc. Med..

[87] Lind A., Totzeck M., Mahabadi A.A., Jánosi R.A., El Gabry M., Ruhparwar A., Mrotzek S.M., Hinrichs L., Akdeniz M., Rassaf T. (2020). Impact of Cancer in Patients Undergoing Transcatheter Aortic Valve Replacement: A Single-Center Study. JACC Cardio Oncol..

[88] Wang T.F., Carrier M., Carney B.J., Kimpton M., Delluc A. (2023). Anticoagulation management and related outcomes in patients with cancer-associated thrombosis and thrombocytopenia: A systematic review and meta-analysis. Thromb. Res..

[89] Amat-Santos I.J., Messika-Zeitoun D., Eltchaninoff H., Kapadia S., Lerakis S., Cheema A.N., Gutiérrez-Ibanes E., Munoz A., Pan M., Webb J.G. (2015). Infective endocarditis after transcatheter aortic valve implantation: Results from a large multicenter registry. Circulation.

[90] Hassanin A., Afify H., Zook S., Frishman W.H., Aronow W.S. (2023). Infective Endocarditis After Transcatheter Aortic Valve Implantation: A Systematic Review. Cardiol. Rev..

[91] Khan A., Aslam A., Satti K.N., Ashiq S. (2020). Infective endocarditis post-transcatheter aortic valve implantation (TAVI), microbiological profile and clinical outcomes: A systematic review. PLoS ONE.

[92] Ghannam A., Gharacholou S.M., Ball C.T., Pollak P.M., Parikh P.P., Landolfo C., Ali M.T., Landolfo K. (2022). Characteristics and Outcomes After Transcatheter Aortic Valve Implantation in Immunocompromised Patients. Am. J. Cardiol..

[93] Murphy A.C., Koshy A.N., Cameron W., Horrigan M., Kearney L., Yeo B., Farouque O., Yudi M.B. (2021). Transcatheter aortic valve replacement in patients with a history of cancer: Periprocedural and long-term outcomes. Catheter. Cardiovasc. Interv..

[94] Zafar M.R., Mustafa S.F., Miller T.W., Alkhawlani T., Sharma U.C. (2020). Outcomes after transcatheter aortic valve replacement in cancer survivors with prior chest radiation therapy: A systematic review and meta-analysis. Cardiooncology.

[95] Kadri A.N., Bernardo M., Assar S.Z., Werns S., Abbas A.E. (2021). Surgical Versus Transcatheter Aortic Valve Replacement in Patients With Malignancy. Cardiovasc. Revasc. Med..

[96] Nashef S.A., Roques F., Sharples L.D., Nilsson J., Smith C., Goldstone A.R., Lockowandt U. (2012). EuroSCORE II. Eur. J. Cardiothorac. Surg..

[97] Durand E., Borz B., Godin M., Tron C., Litzler P.Y., Bessou J.P., Dacher J.-N., Bauer F., Cribier A., Eltchaninoff H. (2013). Performance analysis of EuroSCORE II compared to the original logistic EuroSCORE and STS scores for predicting 30-day mortality after transcatheter aortic valve replacement. Am. J. Cardiol..

[98] Leong D.P., Cirne F., Aghel N., Baro Vila R.C., Cavalli G.D., Ellis P.M., Healey J.S., Whitlock R., Khalaf D., Mian H. (2023). Cardiac Interventions in Patients With Active, Advanced Solid and Hematologic Malignancies: JACC: CardioOncology State-of-the-Art Review. JACC Cardio Oncol..

[99] Krane M., Deutsch M.A., Bleiziffer S., Schneider L., Ruge H., Mazzitelli D., Schreiber C., Brockmann G., Voss B., Bauernschmitt R. (2010). Quality of life among patients undergoing transcatheter aortic valve implantation. Am. Heart J..

[100] Lauck S., Garland E., Achtem L., Forman J., Baumbusch J., Boone R., Cheung A., Ye J., Wood D.A., Webb J.G. (2014). Integrating a palliative approach in a transcatheter heart valve program: Bridging innovations in the management of severe aortic stenosis and best end-of-life practice. Eur. J. Cardiovasc. Nurs..

[101] Turgeon R.D., Ellis U.M., Barry A.R. (2024). Antithrombotic therapy in patients after transcatheter aortic valve implantation: A network meta-analysis. Eur. Heart J. Cardiovasc. Pharmacother..

[102] Arjomandi Rad A., Streukens S., Vainer J., Athanasiou T., Maessen J., Sardari Nia P. (2024). The current state of the multidisciplinary heart team approach: A systematic review. Eur. J. Cardiothorac. Surg..

